# Social and Biological Determinants in Lung Cancer Disparity

**DOI:** 10.3390/cancers16030612

**Published:** 2024-01-31

**Authors:** Briana A. Brock, Hina Mir, Eric L. Flenaugh, Gabriela Oprea-Ilies, Rajesh Singh, Shailesh Singh

**Affiliations:** 1 Department of Microbiology, Biochemistry and Immunology, Morehouse School of Medicine, Atlanta, GA 30310, USA; bbrock@msm.edu (B.A.B.); hmir@msm.edu (H.M.); rsingh@msm.edu (R.S.); 2 Division of Pulmonary Medicine, Morehouse School of Medicine, Atlanta, GA 30310, USA; eflenaugh@msm.edu; 3 Department of Pathology & Laboratory Medicine, Winship Cancer Institute, Emory University School of Medicine, Atlanta, GA 30322, USA; goprea@emory.edu; 4 Cell and Molecular Biology Program, Winship Cancer Institute, Emory University School of Medicine, Atlanta, GA 30322, USA

**Keywords:** lung cancer, disparity, African Americans, socioeconomic status, immunity

## Abstract

**Simple Summary:**

Despite an overall reduction in lung cancer incidence and mortality rates worldwide, African Americans remain disproportionately affected by lung cancer in terms of incidence and outcome, with higher mortality rates than other ethnic or racial groups. Many factors contribute to these racial differences. This review highlights the relationship between social factors and the biology of lung cancer contributing to disparities in incidence and outcome.

**Abstract:**

Lung cancer remains a leading cause of death in the United States and globally, despite progress in treatment and screening efforts. While mortality rates have decreased in recent years, long-term survival of patients with lung cancer continues to be a challenge. Notably, African American (AA) men experience significant disparities in lung cancer compared to European Americans (EA) in terms of incidence, treatment, and survival. Previous studies have explored factors such as smoking patterns and complex social determinants, including socioeconomic status, personal beliefs, and systemic racism, indicating their role in these disparities. In addition to social factors, emerging evidence points to variations in tumor biology, immunity, and comorbid conditions contributing to racial disparities in this disease. This review emphasizes differences in smoking patterns, screening, and early detection and the intricate interplay of social, biological, and environmental conditions that make African Americans more susceptible to developing lung cancer and experiencing poorer outcomes.

## 1. Introduction

Lung cancer is the second most common cancer and the number one cause of cancer deaths in the United States, irrespective of gender, with an estimated 238,340 new cases and 127,070 deaths in 2023 [1]. Notably, African Americans (AA) experience a higher disease burden than any other racial or ethnic group in the United States, as is reflected in higher incidence rates and mortality, as well as a lower likelihood of being diagnosed in an early stage compared to other ethnic groups [2,3]. Conversely, AA women have lower incidence rates than European American (EA) and American Indian women [1]. Smoking cessation has contributed to a steady decline in the incidence rate over the last decade; however, there has been an increase in incidence among non-smokers [1]. Despite awareness and a reduction in smoking, tobacco smoke remains a primary determinant, responsible for 80 to 85% of lung cancer cases and deaths [2]. Non-small cell lung cancer (NSCLC) and small cell lung cancer (SCLC) are the main histological subtypes, accounting for approximately 84% and 13% of all cases, respectively [1,2]. Of all lung cancer cases, 10–25% occur in non-smokers, with almost all being NSCLC subtypes [4,5]. As incidence rates decrease, mortality rates are also slowly reducing due to both smoking cessation efforts and advances in diagnostics and treatment [6]. Early-stage lung cancer usually manifests without symptoms. Most lung nerves belong to the autonomic nervous system; thus, a patient experiences no pain until tumors have grown and the disease has progressed to later stages [7]. Unfortunately, lung cancer places 6th lowest in terms of survival rates among all cancers worldwide, with a 5-year survival rate of 22% in the United States. The 5-year survival rate drops to 8% for advanced-stage cancers [8]. Hence, early detection is key to improving prognosis [9].

A literature search through Pubmed and Scopus on articles published from 2013 to 2023 on lung cancer and factors influencing screening, access to healthcare, treatment and treatment response, tumor biology, and survival was performed. This comprehensive review explores social and biological determinants contributing to disparities in lung cancer incidence and outcome among AAs.

## 2. Smoking Pattern and Lung Cancer Disparity

In addition to variations in incidence and mortality, there are also differences seen in smoking status and behavior, contributing to racial disparities (Table 1). Smoking remains the number one risk factor for lung cancer [8], which is highest among American Indians/Alaskan Natives, followed by AAs, then EAs [10]. In addition to higher rates, AAs are more likely to start smoking at a later age and tend to be lighter smokers than their counterparts [11,12]. Age alone is a risk factor for the development of most cancers. Increased risk of cancer and decreased life expectancy have been associated with the impact of smoking and epigenetic aging [13,14]. Studies have shown that those with a greater number of smoking years are at a higher risk of developing lung cancer compared to those who smoked more packs per year [15,16]. When observing average packs per day, AAs have lower numbers across all age groups but tend to smoke for more extended periods [17]. Hence, smoking patterns contribute to disparities in lung cancer, given that the risk of lung cancer has a stronger correlation with duration than frequency [12,18]. Additionally, choice of cigarettes has been implicated in creating disparate outcomes. Menthol is a cyclic monoterpene alcohol naturally found in mint plants. Since the 1930s, cigarette manufacturers have used it as an additive in tobacco products to reduce the harshness and irritation experienced during smoke inhalation [19]. Menthol cigarettes are associated with a higher dependency than non-menthol cigarettes [11,20]. Studies have shown that this reinforces the effects of nicotine and gives a false perception of being less harmful than non-mentholated cigarettes [18]. AAs smoke menthol cigarettes at a disproportionately higher rate than other groups [18,20].

Studies have also shown a direct association of educational attainment with smoking rates and cessation. Smoking patterns and behavior decrease with education attainment and chances of successfully quitting are higher in the educated population compared to the less educated [12,21]. Irrespective of educational status, AAs are less likely to receive smoking intervention support [20,22]. Additionally, racial bias has been seen in screening and smoking cessation programs, and clinics with more than 50% White patients enrollment are more likely to offer smoking cessation, which is not the case with AAs [23]. The negative impact of smoking on treatment and survival outcomes is well established; however, 50–83% of patients diagnosed with lung cancer continue to smoke [22,24,25]. Compared to other ethnic groups, AAs have lower rates of quitting [22,24,26,27].

## 3. Disparity in Screening and Early Detection Associated with Racial Gap in Lung Cancer Incidence and Outcome

Early detection is a key component in reducing mortality in lung cancer. Imaging methods, such as X-rays, computed tomography (CT) scans, MRIs, ultrasounds, or PET scans and low-dose CT (LDCT) scans, are used to diagnose lung cancer [27,28]. Evidence suggests that mortalities due to lung cancer can be reduced by 20% with annual screening with LDCT compared to X-ray, though LDCT screening utilization sat at a staggering 5% in 2018 [26,27]. Early-stage lung cancer is most often asymptomatic. Therefore, most patients do not show signs or symptoms until advanced stages, contributing to low screening utilization [29]. Previous guidelines set forth by the United States Preventative Services Task Force (USPSTF) recommended LDCT in adults aged 55 to 80 years who have a 30-pack-year smoking history and currently smoke or have quit within the past 15 years [28]. AAs are two-times more likely to develop lung cancer under the age of 50 and tend to have a lower pack-per-year smoking history (25.8 vs. 48) compared to their counterparts [27,28]. Hence, these guidelines failed to capture the most vulnerable populations, particularly AAs, suggesting that race-specific eligibility criteria should be used to increase screening eligibility. Recent guidelines set in 2021 expanded screening criteria by lowering the age from 55 to 50 and the pack per-year minimum from 30 to 20 [28].

Furthermore, lack of access to healthcare and insurance coverage limit the ability of patients to be screened and delay diagnosis, influencing survival. Populations that can afford private insurance are more likely to be screened and/or diagnosed at early stages compared to uninsured patients or those covered by Medicaid [30,31,32]. Since LDCT screening is not cost-effective for insurance companies and Medicaid, screening coverage is only offered to those who meet the screening criteria, and this financial gap leads to lower screening rates in AAs, who are more likely to be medically uninsured, and who have higher rates of insurance loss than EA individuals [33]. The National Cancer Database reported that from 2004 to 2015, 23.57% of AA NSCLC patients that presented with metastatic disease were uninsured or had Medicaid versus 9.66% of EA men. In addition to insurance status, financial barriers negatively impact the lung cancer care continuum. The financial burden resulting from both the direct and indirect costs of cancer treatment is a critical determinant in screening and treatment delays. Expenses such as treatment, transportation and parking, loss of income, medication, and doctors’ visits vary between patients [34,35]. Lung cancer care is more expensive than any other cancer, and a patient’s perception of the cost associated with a positive lung cancer diagnosis influences their decision to undergo screening [35,36,37,38]. 

Provider recommendation for lung cancer screening has a positive impact on screening rates. However, lack of knowledge and lack of provider recommendation have been some of the most common barriers in early detection. The USPSTF recommended lung cancer screening to be grade B in 2013; grade B recommendation means that screening can improve health outcomes and the benefits of being screened outweigh the risks; therefore, clinicians should offer and provide it to eligible patients [28,36]. Nevertheless, utilization remains low; annually, only 15% of eligible adults receive screening. Studies have shown that 12% of patients who could be eligible had conversations with their providers about screening [36,39]. Clinicians’ perceptions and knowledge of available lung cancer screening contribute to a lack of discussion surrounding screening, creating barriers to early detection. For instance, most providers, especially those in rural areas, have insufficient knowledge about screening guidelines, hence the screening recommendation rate is low in rural areas [35,39,40,41,42]. Compared to urban areas, rural areas have historically been underserved and disproportionately burdened with cancer. Although AAs make up less than 10% of rural populations across the United States, disparities in access to healthcare and lung cancer screening still exist between racial and ethnic groups (Table 1) [40]. Proximity to healthcare is better in rural areas with high EA populations compared to areas with low AA populations [43]. Poor outcomes in rural areas could also result from limited access; in rural areas, providers are more likely to have easier access to chest X-rays than LDCT, though chest X-ray is not recommended. Facilities that have access to LDCT are not usually found in rural areas [44,45].

More needs to be achieved to reduce the burden of disease and eliminate disparities in screening. Increasing access to healthcare and reducing financial burdens would help remove barriers to screening and early detection. In addition to reducing barriers, researchers continue to explore other techniques and strategies, such as using biomarkers for the early detection and diagnosis of lung cancer [46]. Other efforts to increase utilization involve partnerships between investigators and communities implementing outreach programs to educate and inform citizens about lung cancer, care, and screening [28,47].

## 4. Association of Social Factors in Lung Cancer Disparity

Socioeconomic status (SES) is a combined social and economic measure of a person’s education, income, occupation, living conditions, and access to opportunities and resources. SES has been recognized as a key determinant of all-cause mortality, influencing a person’s health throughout their lifespan [23,48,49,50,51]. Moreover, among all cancers, SES has a big impact on lung cancer and the observed disparity in incidence, treatment, and outcome (Table 1) [52]. When accounting for sex, the disparity in lung cancer risk is more apparent among men [52,53]. Several studies have shown an association between better quality of life and higher SES among patients diagnosed at early-stage lung cancer (i.e., stage I/II) [54]. To measure the impact on health outcomes, SES can be assessed at various levels, such as personal, interpersonal, and societal levels [48,50,51,55]. AAs tend to have lower economic status than all other ethnic groups in the United States. The results from the United States Census Bureau illustrate that AAs had the lowest levels in all indicators of SES (median household income, median wealth, non-home wealth, and home ownership) among all ethnic groups, with the exception of education obtained wherein Hispanic people had lower rates [21]. People with lower SES tend to have lower levels of education and economic development and live in areas with fewer resources than those with higher SES [21]. Lung cancer incidence, screening, survival, treatment options, and treatment choice are associated with education and income levels. Higher education levels and socioeconomic status are associated with lower smoking rates [56]. A study using data from the National Survey on Drug Use and Health suggested that 38% of gaps in smoking prevalence across different racial and ethnic groups were attributed to variations in education level [12]. Another study determined that across the globe, the odds of smoking were 69% higher in lower-class than in middle-class individuals [57]. This falls in line with other studies that have strongly correlated SES with smoking [12,56]. In addition, individuals with lower SES have higher levels of mistrust in society than others, influencing healthcare utilization and decision making. Collectively, these perpetuate the disparities in lung cancer incidence and survival rates observed in AAs [55].

**Figure 1:**
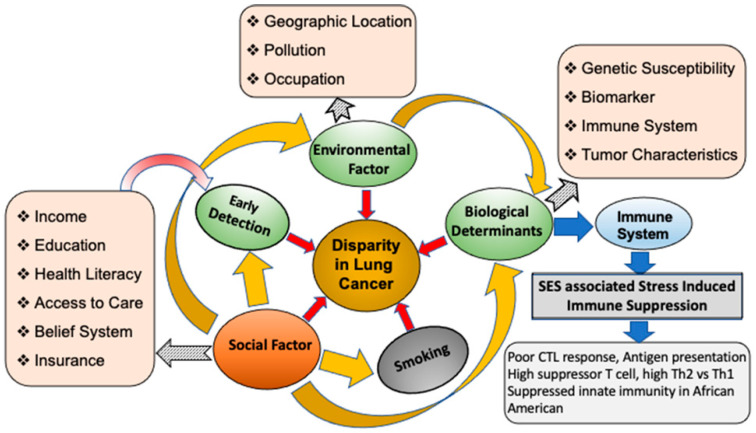
Social and biological determinants of lung cancer. The diagram shows an association of social factors such as income, education, health literacy, access to care, belief system, and insurance status with racial differences in lung cancer incidence and outcome. Low socioeconomic status negatively impacts education, income, health awareness, and access to insurance, leading to lower screening rates, unhealthy geographical location, forced choice of occupation, and frequent proneness to exposure to hazards. Low SES also induces chronic stress, impacting the biology of lung cancer and favoring an immunosuppressive environment in African Americans.

Evidence has shown a strong association between SES and academic achievement. The National Center for Education Statistics conducted longitudinal studies showing that household SES plays a role in post-secondary enrollment and employment [58]. Children in lower SES households tend to have poorer cognitive development and socioemotional skills than those in households with higher SES. Both cognitive development and socioemotional skills have been identified as predictors of upward social and economic mobility [58]. Schools in areas with low SES have fewer resources, less funding, and higher dropout rates than schools in higher-SES areas, directly impacting academic achievement and employment [59]. Students with lower SES were 50% less likely to enroll in post-secondary education and complete it. Furthermore, children from higher SES households are more likely to attend better post-secondary institutions [58]. One study showed that education level may be a more appropriate way to determine how information is disseminated to patients with cancer [60]. Educational attainment provides opportunities for better job opportunities, thus improving income. Data from the Bureau of Labor Statistics show evidence that educational attainment is directly related to median income, with 4-year college graduates earning 84% more than high school graduates. Even with similar education and occupation levels, AAs earn less than both Asian and EA populations. EAs with bachelor’s degrees tend to earn more than AAs with master’s degrees [21]. This increases racial inequalities by hindering the economic development and socioeconomic mobility of AAs (Figure 1). 

There has been a direct correlation between increasing median income and better health across the United States. Zip codes with lower SES correlated with heavier disease burdens, including cancer [49]. Economic growth is instrumental in reducing poverty and leading to a better quality of life. The mortality rate in lower median household incomes is 28% higher than in areas with higher median incomes [49]. Furthermore, those in rural areas have higher mortality rates than those in urban areas. Irrespective of rural or urban areas, the 5-year is better in EAs than any other ethnic group, including AAs [61]. The availability of resources such as hospitals, specialists, and other medical facilities has been associated with area-level SES. Access to quality and preventative care increases the likelihood of “healthy aging” [49,62]. However, access to these resources is limited in rural areas and low-SES areas. The cost of living is higher for people with middle-to-low SES; therefore, they are less likely to seek treatment when funds and resources are unavailable or their basic needs are unmet [63,64]. Due to low SES, they are less likely to take sick days away from work and are more likely to work through illnesses when compared to people in higher SES groups [64], which contributes to a low lung cancer screening rate, late diagnosis, and high mortality due to lung cancer in low-income populations, specially in AA population [48,49,61]. In addition to late diagnosis and poor survival, studies have shown an association between SES and lung cancer survival (Table 1; Figure 1) [54]. In contrast to this, one study revealed that disparities in survival rates did not exist between patients from different socioeconomic backgrounds following their clinical trial participation [65]. However, the study only included 5724 AA and 65,449 EA patients [65]. 

The Center for Disease Control (CDC) defines health literacy as “the degree to which individuals can obtain, process, and understand basic health information and services needed to make appropriate health decisions”. Studies have shown that low health literacy is not uncommon and tends to be lower amongst people with low SES and low education attainment, causing a potential barrier to appropriate education and decision making regarding lung cancer screening and treatment [23,66]. Health literacy is important for informed clinical decision making and has been correlated with patient outcomes, and AAs are two-times more likely to have basic or below-basic health literacy compared to EAs [23]. 

Systemic racism contributes to disparities in health and health outcomes. Historically, discriminatory policies, redlining, racial segregation, and abuse by researchers and healthcare providers have deprived minorities of access to education, jobs, quality healthcare, and trust [23,48]. Redlining is a form of discrimination by denying resources and services to people in neighborhoods deemed “risky” or “hazardous” for investments. Historically, redlining was used in government-funded homeowner programs to preserve segregation by denying home loans to people in areas that were considered extremely risky. Most AAs lived in these areas, forcing them to remain in areas with low SES and limited geographic mobility [67]. Studies were conducted to examine the association between breast cancer outcomes and patients living in historically red-lined areas. The majority of these were conducted at state or county levels and only in breast cancer patients, limiting generalizability. However, they agreed that there were higher rates of late-stage diagnosis, lower screening rates, and an increased prevalence of chronic conditions associated with residence in redlined areas compared with patients who did not live in those areas. In the context of lung cancer, AA men residing in redlined areas in Boston were 47% less likely to undergo screening compared to EA men in redlined areas. Additionally, studies have shown that stress levels strongly correlate with SES. Populations with lower education and income tend to have higher levels of stress [68,69].

## 5. Association of Environmental Factors with Lung Cancer Disparity

Environmental exposures in both living and occupation settings have been implicated in increasing the risk of lung cancer and contributing to disparity (Table 1). Environmental pollutants such as CO, NO_2_, CO_3_, and small particulate matter can lead to many health complications, including respiratory diseases and increased cancer risk [48,70]. A study in California showed an association between traffic pollution and lung cancer risk, noting that AAs were disproportionately affected. Populations with low SES tend to live in environments that make them prone to more adverse health outcomes. Particularly, AAs tend to live in areas in closer proximity to industrial sites, placing them at greater risk for cancer and potentially increasing low-grade inflammation [48,71]. These sites include waste disposal, power plants, superfund sites, and other hazardous sites. Superfund sites have been historically associated with increased risk and incidence of lung diseases such as COPD and lung cancer [72]. An environmental justice group reported that 70% of all superfund sites were located within 1 mile of federally funded housing, disproportionately affecting AAs [48]. Strikingly, Mikati et al. evaluated point-source particulate matter across the United States and found that not only were exposures to particulate 35% more likely in low SES communities when compared to high SES communities, but within these communities, AAs were 54% more likely to be burdened than EAs [73].

Studies analyzing the impact of occupational status and esteemed professions have shown that occupations with lower prestige increase the risks of lung cancer [74]. Some of these differences can be explained by occupational exposure and smoking behaviors [74,75]. Occupational exposures such as asbestos, diesel exhaust, coal mining, painting, chimney sweeping, and paving with coal tar exposure increase the risk of developing lung cancer, and populations with low SES are often engaged in these jobs [74,76,77]. Non-smokers working in these industries have an increased risk of lung cancer, whereas smokers within these fields have a significantly higher risk [76,77]. A combination of educational attainment and low SES limit occupational opportunities, contributing to disproportionate numbers of AAs representing the workforces that are exposed to industrial hazards, as is reflected in higher risks of occupational exposure to carcinogens. These jobs usually do not have education requirements and are labor intensive. Even within the same jobs, AAs are more likely to encounter hazardous exposures when compared to EAs [78]. These exposures have been known to cause lung tissue injuries resulting in inflammation, increasing the risk of cancer development [79].

## 6. Racial Disparities in Lung Cancer Treatment

Differences in biology also significantly contribute to disparities in incidence, treatment, disease progression, and survival outcomes. Treatment regimens for lung cancer include surgery, radiation, chemotherapy, targeted therapies, and immunotherapy. The treatment plan largely depends on patients’ overall health, histological subtype, and stage at diagnosis. Patients diagnosed at an early stage (stage I or II) are more likely to undergo surgery than if diagnosed at later stages [80], but only 55% of those diagnosed at an early stage undergo surgery. AAs are less likely to undergo surgical resection irrespective of the stages at which they are diagnosed [2,81]. Those who opt-out of surgery or cannot receive it often receive radiation therapy. Targeted therapy is considered the standard of care for patients diagnosed at later stages with targetable mutations. Even so, a combination of chemotherapy, radiation therapy, and surgery remains an option. A key component of targeted therapy and personalized medicine includes biomarker testing and molecular profiling. In contrast, patients who lack targetable mutations are usually recommended immunotherapy as a first-line treatment. Immunotherapy aims to potentiate a patient’s immune system to fight lung cancer by targeting immune evading mechanisms of the tumor. Current immunotherapies include cancer vaccines, cytokine therapies, oncolytic virus therapies, immune checkpoint inhibitors, and adoptive cell transfer. For instance, immune checkpoint inhibitors, such as anti-programmed death ligand 1 (anti-PD-L1), anti-PD-1, and cytotoxic T-lymphocyte associated antigen-4 (CTLA-4), are monoclonal antibodies used to target immune checkpoints [82]. These inhibitors reinstate patient’s immune surveillance and break cancer cell induced immune tolerance by activating or inhibiting appropriate T lymphocyte (T-cell) responses [82,83,84]. These therapies are used together to treat NSCLC and enhance immune response [83,85,86,87,88].

In cases where patients are unable to undergo surgery or utilize targeted and/or immunotherapy, chemotherapy remains an option. Histological differences and specific driver mutations affect therapeutic efficacy. Despite recent advances in treatment, differences exist among treatment patterns, exacerbating disparities in survival. AAs diagnosed with stage I/II are less likely to undergo surgery and are more likely to remain untreated when compared to EAs [2]. AAs are also less likely to receive EGFR testing and erlotinib, a common EGFR inhibitor when compared to all other ethnic groups. They are 20% less likely to ever receive biomarker testing than EAs. NGS testing influences participation in clinical trials. The probability of being included in clinical trials is twice as likely to occur with NGS testing; unfortunately, AAs are 55% less likely to be included [89]. Only 27% of cancer patients have access to clinical trials. Historically, EA men are overrepresented in studies. Only 7% of enrolled participants in clinical trials resulting in FDA approval were AA [90]. Hence, less effective treatment options are available to AA patients primarily because of a lack of clinical trial data due to their underrepresentation in FDA-approved clinical trials (Table 2) [91,92,93]. 

### 6.1. Tyrosine Kinase Inhibitors and Disparity in Treatment Outcome of Lung Cancer

Epidermal growth factor (EGF) receptors (EGFR), also known as HER1/ErbB1, are members of the HER/ErbB receptor tyrosine kinase family proteins. These are transmembrane growth factor receptors with tyrosine kinase activity. EGFR is commonly expressed in most cells including all stromal and epithelial cells. There are seven ligands that bind to EGFR: EGF, transforming growth factor-alpha (TGF-α); heparin-binding EGF-like growth factor (HB-EGF); epigen; epiregulin; amphiregulin; and β-celluin. These ligands elicit different responses and affect pathways such as Ras/Raf/MAPk, PI3K/Akt/mTOR, and JAK/STAT [84,94,95]. Therefore, gene amplification and gain-of-function mutations within the tyrosine kinase domain cause EGFR to become oncogenic. Smoking status, ethnic background, and gender have impacts on the prevalence of EGFR mutations. Overexpression of EGFR is found in 40–89% of lung cancer patients and mainly in adenocarcinomas. There has been a strong association between non-smoking and EGFR mutations. Asians have a higher prevalence than EAs and AAs; women tend to have a higher prevalence than men [84,94,95,96,97,98,99,100,101]. Treatments targeting mutated EGFR, including anti-EGFR monoclonal antibodies (mAbs) and tyrosine kinase inhibitors (TKIs), are offered to treat lung cancer. Anti-EGFR mAbs bind to the extracellular domains of EGFR, blocking ligands from binding and promoting receptor internalization, thus preventing intracellular signaling. Tyrosine kinase inhibitors (TKIs) aim to inhibit the signaling of tyrosine kinase domains by targeting genes such as EGFR, ALK, HER2, and ROS1 [83,102,103,104]. TKIs are ATP analogs that competitively bind in ATP pockets on the intracellular kinase domain of the receptor, thus inhibiting its autophosphorylation and, ultimately, intracellular signaling. Autophosphorylation is a key component in regulating enzymatic activity and promotes the binding of other signaling molecules [84,105]. Patients with EGFR mutations have longer survival than those with KRAS mutations. Even so, studies have shown that AAs harboring EGFR and/or KRAS mutations in NSCLC have shorter 2-year survival rates (33%) than EAs (32%) with similar mutation frequencies [106].

### 6.2. KRAS and Disparity in Treatment Outcome of Lung Cancer

The Kirsten rat sarcoma viral oncogene homolog (KRAS) gene codes for the k-ras protein. K-ras, a member of the RAS superfamily, is a GTPase transducer protein. RAS proteins remain inactivated until they bind to GTP. KRAS gene mutations produce active forms of k-ras, resulting in an upregulation of cell growth and survival via the Raf/MAPk and PI3K pathways. KRAS mutations drive 35% of all lung cancers and are associated with patients who are current or previous smokers; however, due to the prevalence of these mutations in non-smokers, smoking status is a poor predictor of mutational status in NSCLC. The k-ras protein has been deemed an “undruggable” target due to its smooth surface and lack of pockets, making it difficult to target. Researchers have been working to target molecules upstream and downstream of the molecule, such as TKIs. One promising therapy targets a specific mutant k-ras protein (G12C). KRAS mutations have also been associated with EGFR TKI resistance [84,107,108]. Furthermore, the prevalence of KRAS mutations is higher in Western countries than in Asian countries, despite similar smoking patterns [96,99,107,109]. A few studies have shown that the prevalence of KRAS mutation is lower in AAs than in EAs; however, smoking status was not taken into consideration, and sample sizes were small [110]. Besides, this was in contrast to other studies that have shown no difference or a higher prevalence in KRAS mutations with smoking [106].

## 7. Racial Differences in Lung Cancer Biology Contributing to Disparity

Emerging studies have shown that tumor biology varies across races, contributing to disparities seen in incidence rates, mortality rates, and treatment response. Allostatic load and social adversity are correlated with increases in pro-inflammatory signaling, resulting in immune suppression, which accelerates cancer progression. The intersectionality of social factors leads to a higher disease burden among AAs (Figure 1). Race-based differences in tumor biology and adaptive and innate immunity pose challenges for treatments and outcomes.

### Racial Difference in the Immunological Landscape Contributes to Lung Cancer Disparity

Differences in adaptive and innate immunity between ethnic groups create variations in immune responses, cancer progression and metastasis, and response to treatment [25,111,112]. Innate immunity is the body’s first defense against invading pathogens. Adaptive immunity is stimulated when the innate system fails to eliminate pathogens [113]. The process of inflammation, when the body responds to pathogens and damage to cells and tissues, has been closely linked to cancer initiation and progression, impacting innate and adaptive immunity [114,115]. Evidence has shown that low-grade and chronic inflammation plays a role in the initiation, progression, and metastasis of most cancers [113]. Cytokines that promote the growth and proliferation of normal cells also promote cells that contain mutations [115]. Crosstalk between tumor cells, immune cells, and the surrounding stromal cells dictates whether pro-tumor or anti-tumor immune mechanisms occur [69]. Tumors owing to their heterogenous, and adaptive characteristics create favorable microenvironments by recruiting suppressive immune cells, such as regulatory T-cells, which inhibit the functions of NK cells, macrophages, DCs, and CD4 and CD8 T-cells, promoting tolerance. Increased levels of pro-inflammatory cytokines and regulatory T-cells have been associated with poor survival outcomes [115]. 

Stress has been shown to suppress the immune system against cancers including lung cancer and is also linked to conditions such as hypertension, diabetes, and other chronic illnesses affecting AAs at disproportionate rates [48,69]. As mentioned previously, AAs tend to have higher levels of stress and stress-associated disease, producing higher levels of stress hormones, such as cortisol, catecholamine, epinephrine, and norepinephrine [48,71,116]. Higher cortisol and catecholamine levels can suppress immune function and hinder immune cell trafficking, respectively [117]. This is important to consider, as many comorbid diseases with higher prevalence among minority ethnicities implicate hormone dysregulation [48,71]. Studies have shown that AAs have higher levels of IL-6 and regulatory T-cells in circulation and within the tumor microenvironment when compared to other ethnic groups, which is an indicator of an immunosuppressive tumor environment (Figure 1) [106,118,119]. IL-6 levels have been shown to increase with tumor stage and during lung cancer progression, and have been associated with poor survival [120]. A case–control study following recently diagnosed patients aimed to determine if stressful life events were associated with diagnosis. This study found that those with the disease were 78% more likely to have experienced a stressful life event 5 years prior to their clinical diagnosis [121]. Taken together, stress levels due to lower SES create more favorable environments for lung cancer development and progression (Table 2) [68,121,122].

## 8. Association of Comorbid Conditions with Disparity in Lung Cancer Outcome

Comorbidities can induce cancer and cause adverse tumor biology, leading to more aggressive cancer. Lung cancer patients with comorbidities have higher mortality rates than individuals who do not [92,123]. In fact, these conditions impact treatment options and effectiveness. Studies have shown that individuals with co-morbidities are less likely to receive aggressive and/or curative options than those without them [124]. AAs are disproportionately affected by chronic kidney disease, coronary heart disease, hypertension, obesity, and diabetes [92]. Many of these comorbid diseases have been associated with stress and low-grade inflammation, contributing to disparate incidence and more aggressive disease, resulting in poorer outcomes [123,125]. The presence of comorbid conditions disqualifies a patient from receiving most of the available treatments. In addition, exclusionary criteria for clinical trials include comorbidities that disproportionately affect AAs, excluding them from participation. 

## 9. Conclusions

African Americans continue to face a disproportionate burden of lung cancer, experiencing disparity in incidence, treatment, and outcomes. The persistence of systemic and structural racism contributes significantly to these inequities, linking them to various factors affecting racial and ethnic groups (Figure 1). Addressing these issues necessitates efforts to enhance healthcare and insurance access, potentially improving screening rates and early detection. Recommendations from the U.S. Preventive Services Task Force (USPSTF) should be adjusted to be more inclusive of minority groups and vulnerable populations currently overlooked in screening.

Considering variations in smoking patterns, it is recommended to revise lung screening guidelines to include considerations for race and ethnicity. This adjustment aims to ensure early identification of individuals in the initial stages of lung cancer, potentially boosting overall survival rates. Despite adjustments for social factors, persistent disparities underscore the impact of biological differences in lung cancer outcomes. Limited and conflicting data on mutations associated with lung cancer among different racial and ethnic groups, smokers and non-smokers, and individuals with varying socioeconomic statuses highlight the need for more extensive studies with diverse and adequate sample sizes.

To address knowledge gaps and develop better interventions for lung cancer, it is imperative to understand the intersectionality of biological differences and the influence of social and environmental factors. This is highlighted in Table 1 and Table 2, emphasizing the importance of comprehensive research in this domain (Table 1 and Table 2).

## Figures and Tables

**Table 1:** Multidimensional effects of social determinants of health on lung cancer burden in African American men.

Social and Lifestyle Factors	Lung Cancer Predisposing Effects	Status in the African American Population
Smoking habit	➢ Smoking increases the risk of the development of lung cancer.	➢ AAs tend to smoke fewer packs per day but for a greater number of years. This pattern of smoking increases the risk of lung cancer. ➢ Lack of support to help facilitate cessation. ➢ AA smokers are more inclined to menthol cigarettes, which are more addictive than non-mentholated cigarettes, than other racial groups
Lower Socioeconomic Status	➢ Higher stress levels have a negative impact on the immune system and have been associated with many comorbid conditions. ➢ Increased barriers to healthcare lowering the likelihood of seeking medical treatment: ∘ Higher rates of uninsured/underinsured individuals; ∘ Lower rates of insurance utilization; ∘ Delayed diagnosis results in late-stage diagnosis, which is often combined with delayed treatment, contributing to disparities observed in stage-at-diagnosis, treatment, and outcomes.	➢ African Americans (AAs) are more likely to have lower socioeconomic status compared to European Americans (EAs), reflected in both income and wealth. ➢ AAs frequently suffer from comorbidities which limits their participation in lung cancer clinical trials. ➢ AA insurance status is a major determinant in receiving lung cancer screening, genetic and molecular testing, and guiding treatment.
Poor Education Level	➢ Low health literacy impacts decision making regarding screening and treatment. ➢ Lower economic mobility and SES. ➢ Limited occupational opportunities. ➢ Higher stress levels affect overall health. ➢ Higher rates of smoking.	➢ AA men tend to have lower educational levels than EAs.
Occupational Hazards	➢ Industrial pollution and hazards (such as diesel exhaust, coal tar, and asbestos) are known to increase the risk of lung diseases and cancer. ➢ These occupations have also been associated with higher stress levels.	➢ AA men are more likely to be exposed to industrial hazards and pollution when compared to EAs due to limited job prospects.
Choice of Geographic Locations	➢ Environmental pollution and waste from industrial and superfund sites have been implicated in many types of cancer, including lung cancer. ➢ Inadequate or no access to healthcare resources impacts timely lung cancer screening, diagnosis, and treatment. Altogether these limit treatment options and decrease overall survival. ➢ Rural areas when compared to urban areas are more likely to have: ∘ Higher rates of comorbid conditions; ∘ Limited access to medical care; ∘ Lower rates of insurance; ∘ Higher rates of smoking; ∘ Lower levels of SES; ∘ Lower rates of surgery and treatment; ∘ Increased disparities.	➢ AAs tend to live in areas with lower SES which are more likely to be near superfund sites, industrial sites, and areas with increased environmental pollution compared to areas with higher SES. ➢ Rural areas with a higher density of AAs are more likely to experience hospital closures when compared to areas with lower AA population density.

**Table 2:** Clinical and biological factors underscore the higher incidence and poorer outcomes in African American lung cancer patients.

Clinical and Biological Factors	Impact on the Treatment Regime	Concerns Specific to African American Lung Cancer Patients
Incidence	➢ Detection at an early stage gives more treatment opportunities and improves the chances of survival of lung cancer patients.	➢ AA men are diagnosed in later stages when the disease has progressed much further as compared to EA lung cancer patients. ➢ Late-stage lung cancer diagnosis results in more aggressive disease and, hence, lower survival rates in AA men compared to EAs. ➢ Low-grade chronic inflammation in AA men may be predisposing them to earlier precipitation.
Comorbid Conditions	➢ Very commonly, comorbidities disqualify a patient from participating in lung cancer clinical studies.	➢ Due to SES-associated stress, AAs disproportionately suffer from comorbid conditions such as hypertension, kidney disease, and diabetes when compared to EAs. ➢ This factor not only negatively affects the treatment outcome, but also limits their participation in clinical trial studies oriented to understanding and developing treatments for lung cancer.
Mutational make up of lung tumors	➢ Precision medicine relies on understanding genetic differences to target cancer.	➢ Few studies provide race-specific information on lung cancer-associated mutations. ➢ Further, these studies provide conflicting evidence regarding the prevalence of targetable mutations in AAs, largely due to the lack of ethnic variation in conducted research or small sample sizes.
